# Obsessive-compulsive disorder is a heterogeneous disorder: evidence from diffusion tensor imaging and magnetization transfer imaging

**DOI:** 10.1186/s12888-015-0535-5

**Published:** 2015-06-25

**Authors:** Alexander Glahn, Tino Prell, Julian Grosskreutz, Thomas Peschel, Kirsten R. Müller-Vahl

**Affiliations:** 1 Department of Psychiatry, Social Psychiatry and Psychotherapy, Hannover Medical School, Hannover, Germany; 2 Department of Neurology, University Hospital Jena, Jena, Germany

**Keywords:** Magnetization transfer imaging, Fractional anisotropy, Apparent diffusion coefficient, Diffusion tensor imaging, Obsessive-Compulsive disorder, Parietal cortex

## Abstract

**Background:**

Current models of obsessive compulsive disorder (OCD) propose abnormalities of cortico-striatal circuits that involve the orbitofrontal cortex, anterior cingulate cortex, thalamus and the striatum. Nevertheless, during the last years, results of morphometric studies were contradictory. Since fully automated whole-brain voxel-based morphometry (VBM) and diffusion tensor imaging (DTI) are used to assess structural changes in OCD patients, increased consistent evidence has been reported that brain abnormalities are not limited exclusively to the “affective” orbitofronto-striatal circuit. Moreover, several studies conducted using a symptom dimensional approach demonstrated that different symptoms are mediated by distinct neural systems.

**Method:**

We investigated structural brain abnormalities in 14 carefully selected adult (≥18 years), male and unmedicated patients with OCD - separately for obsession and compulsion scores (Y-BOCS) - compared to 20 healthy controls as reflected according to white matter changes by fractional anisotropy and apparent diffusion coefficient. Moreover, this is the first study in OCD patients, using magnetization transfer imaging (MTI). This method is said to be more sensitive to subtle structural brain changes than conventional volumetric imaging.

**Results:**

In our study, we show a positive correlation between MTR and Y-BOCS obsession scores with an increased integrity of tissue structure in the parietal cortex, including myelination and axonal density reflected by the magnetization transfer ratio (MTR) which was used for the first time in our study. Furthermore, Y-BOCS scores for compulsions correlated negatively with ADC-maps in the left nucleus lentiformis and the cingulum.

**Conclusion:**

The results support the hypothesis that OCD is a heterogeneous disorder with distinct neural correlates across symptom dimensions and call for a substantial revision of such a model that takes into account the heterogeneity of the disorder.

## Background

Obsessive-compulsive disorder (OCD) is a disabling anxiety disorder with a lifetime prevalence rate of 2–4 % in the general population. It is characterized by the presence of intrusive and distressing thoughts, images or ideas, termed obsessions and repetitive or ritual behaviours, known as compulsions [1]. However, the underlying neurobiological mechanisms are still unknown. Previous neuroimaging studies have demonstrated that changes of white (WM) and grey matter (GM) in several brain regions seem to be involved in the pathogenesis of OCD. Current models of OCD propose abnormalities of cortico-striatal circuits that involve the orbitofrontal cortex (OFC), anterior cingulate cortex (ACC), thalamus and the striatum. Nevertheless, during the last years, results of morphometric studies were contradictory [2–11]. For example, there are reports of increased GM volume in the OFC and other structures belonging to the orbitofronto-striatal circuit [12–14], but also of reduced volumes in the same regions [3, 15] or even of no changes [16].

Diffusion tensor imaging (DTI) studies have investigated WM abnormalities in OCD patients compared to healthy controls, showing abnormalities of the fronto-striatal thalamo-cortical loop [17, 18]. The cingulate bundle, the corpus callosum and the anterior limb of the internal capsule seem to be most commonly affected by decreased white matter integrity in adult OCD patients [18]. Within cortical structures, higher fractional anisotropy (FA) and apparent diffusion coefficient (ADC) were observed in the medial frontal region [2, 19] and lower FA was found in the parietal lobe, left lingual gyrus and occipital lobe white matter [17]. A review by Koch et al. on DTI studies in OCD showed that the cingulate bundle, the corpus callosum and the anterior limb of the internal capsule seem to be most commonly affected by decreased white matter in adult OCD patients [18]. In the cingulum, lower FA was reported in the bilateral anterior [20] and right posterior cingulate gyrus [17]. However, Cannistraro et al. [21] showed greater FA values in the left, but lower FA values in the right cingulum bundle. Regarding the corpus callosum, Yoo et al. [22] found higher FA in the corpus callosum, while Nakamae et al. [19] found smaller FA in the anterior body of the corpus callosum.

It can be assumed that these inconsistencies of GM and WM abnormalities in previous studies can mainly be attributed either to substantial methodological differences or to the inclusion of only small and heterogeneous samples. Since fully automated whole-brain voxel-based morphometry (VBM) and DTI are used to assess structural changes in OCD patients, it has been consistently reported that brain abnormalities are not limited to the “affective” orbitofronto-striatal circuit but also extend to the dorsolateral prefronto-striatal “executive” circuit and additional regions including the parietal and occipital lobes as well as the cerebellum [2, 6, 7, 23–25]. Another important source of variability is the clinical heterogeneity of OCD. It is becoming increasingly clear that OCD is not a unique disorder, but consists of multiple potentially overlapping symptom dimensions [26]. Despite increasing recognition of this phenotypic heterogeneity, according to standard nomenclatures (such as DSM-IV-TR and ICD-10) OCD is still considered a unitary nosological entity [7]. However, patients diagnosed with OCD widely vary according to symptom type (compulsions vs. obsessions), different kinds of obsessions and compulsions (e.g., hoarding vs. cleaning), severity, age of onset, comorbidities (e.g. tics, depression) and medication as an influencing factor [7]. Therefore, recent studies used a symptom dimensional approach [27] and were able to demonstrate that different symptoms are indeed mediated by distinct neural systems [6, 28, 29].

The aim of this study was to further investigate structural brain abnormalities in carefully selected adult (≥18 years), male and unmedicated patients with OCD compared to healthy controls as reflected according to WM changes by FA and ADC. Moreover, this is the first study in OCD patients, using magnetization transfer imaging (MTI). Magnetization transfer imaging (MTI) detects the relative proportion of free mobile protons and immobile protons bound to macromolecules. Protons that are bound to macromolecular structures, such as myelin are characterized by restricted motion while protons in free water have relatively unrestricted motion and the interaction between protons in these two pools produces the contrast that allows for tissue differentiation. Here we used MTI because it is capable of detecting subtle neuropathological changes *in vivo* before it manifests on conventional MR volumetric imaging [30]. A decrease of MTR can be observed in several pathological processes, such as multiple sclerosis or stroke conventional. On the other hand an increase of MTR is associated with brain myelination, probably as a result of increasing interactions between “bound” water and glycolipids, cholesterol, and galactocerebrosides in myelinating neurons [31].

## Methods

### Subjects

In this study, 14 unmedicated (drug free for at least 1 year) male patients with OCD (mean age = 39.9 years, range, 21.8–65.1) according to DSM-IV-TR criteria and 20 age- and sex-matched healthy control subjects (mean age = 31.7, range, 18.2–65.4, *p* = 0.71) were enrolled. All patients were investigated by one of the authors (KRMV), who is experienced in the diagnosis of OCD. For assessing the severity of obsessions and compulsions the German version of the Yale-Brown obsessive compulsive scale (Y-BOCS) was used [32, 33]. Accordingly, 12 patients were diagnosed with OCD with combined obsessions and compulsions (Y-BOCS > 16). Two patients suffered either from compulsions or obsessive thoughts (Y-BOCS ≥ 10) [32, 33]. Clinical characteristics and scores are summarized in Table 1. None of the patients fulfilled diagnostic criteria for comorbid depression, attention deficit hyperactivity disorder (ADHD), anxiety disorder, or Tourette syndrome according to DSM IV-TR as assessed by a clinical interview and the Beck’s Depression Inventory-II (BDI) [34], the State-Trait Anxiety Inventory (STAI) [35], the short form of the German version of the Wender Utah rating scale (WURS-k) [36] and the Yale Global Tic Severity Scale (YGTSS) [37]. None of the patients had a history of head trauma, epilepsy, brain surgery, systemic illness, or drug or alcohol abuse. Physical and neurological examination and routine blood laboratory tests were normal. Healthy controls were interviewed and examined in the same way as patients. All patients and controls were right handed. This study was approved by the local ethic committees of the Hannover Medical School and was carried out in accordance with the declaration of Helsinki. All participants gave written informed consent after all procedures had been fully explained to them before entering the study. All participants provided consent for the publications of anonymized individual clinical details (Table 1).

**Table 1:** Clinical characteristics and Y-BOCS-Scores of the OCD patients

Patient	Age	Subscores Obsession	Subscores Compulsion
1	29	15	16
2	40	16	18
3	50	0	18
4	65	12	0
5	42	15	16
6	35	17	13
7	20	19	17
8	63	13	12
9	21	17	18
10	43	19	11
11	38	11	10
12	52	16	16
13	21	12	14
14	32	17	12
Mean	39,96	14,21	13,64
SD	14.80	4,99	4.78

### Data acquisition

Images were acquired on a neuro-optimized 1.5-T GE Signa Horizon LW (General Electric Company, Milwaukee, WI, USA) using a 3-dimensional T1-weighted spoiled gradient recalled echo (SPGR) sequence generating 124 continguos sagittal slices (RT 24 ms; TE 8 ms; flip angle 30°, 2 averages, acquisition time 13′10″, in plane resolution 0.97 × 0.97 ×1.5 mm^3^). The protocol for the MTI consisted of a proton density (PD)-weighted SE sequence (TR 2600, TE 20, 256 × 256) both with (MT) and without (non-MT) a preparing saturation pulse (1200 Hz off-resonance, 1180° flip-angle, 16 ms). 48 slices of 3 mm thickness were acquired. Image post-processing included a simple intersequence correction of movement with the automated image registration package based on rigid body model (AIR) and calculation of MTR maps pixel-by-pixel according to the following formula: MTR = ([non-MT – MT] / non-MT) × 100. DTI was performed using echoplanar imaging (EPI) (39 contiguous slices, 3 mm thickness, 2 × 2 mm in plane resolution, 24 directions, *b* = 1000, total scanning time 25 min). One *b* = 0 image was obtained. During scanning, all participants were comfortably placed and their heads were fixated within the headcoil with special cushions. All subjects received additional T2-weighted images.

### Pre-processing

Data were processed on a standard IBM-compatible PC using SPM2 statistical parametric mapping software (Welcome Department of Cognitive Neurology, London) and working in an analysis environment (version 6.1; the Math Works Inc, Natick, Mass). Images were reoriented into oblique axial slices aligned parallel to the anterior-posterior commissural axis with the origin set to the anterior commissure.

After calculating the FA and ADC maps, images were pre-processed and analyzed by SPM2 using an approach adopted from VBM. This included an optimized normalisation procedure, together with an automated exclusion of skull and CSF signal values and smoothing (8-mm FWHM). In a first step, all EPI scans were normalized to the EPI-template provided by SPM. The normalized data were then smoothed (8-mm FWHM) and a mean image was created. This provided an EPI site- template appropriate to the population sample and with scanner specific image contrast. In a second step, all EPI images in native space were then normalized to the template derived from step 1. As statistical inference was intended to be only made on the intensities of brain tissue, the individually normalized images were cleaned to remove extracerebral tissue and CSF. This was accomplished by using the segmentation function of SPM2 including the brain extraction step on the normalized images. The white and grey matter partitions were then reunited using the ImCalc function. The resultant image was thresholded by 0.15 to create a binary mask image containing ‘ones’ for voxels with a probability of greater than 15 % to belong to grey ore white matter and ‘zeros’ for voxels outside the brain. The mask image was then multiplied voxel-by-voxel with the corresponding normalized EPI image, thus discarding the majority of extracerebral tissue and CSF and preserving the original voxel intensities. Those cleaned normalized images were then smoothed and a mean image of all subjects – the cleaned EPI template - was created. In a third step, the same cleaning procedure was applied to the EPI images in native space. The resultant cleaned images were then normalized to the cleaned template from step 2 thus preventing any contribution of nonbrain voxels and affording optimal spatial normalization of the cleaned EPI images. The optimized normalisation parameters were reapplied to the coregistered FA images. This resulted in optimally normalized fractional anisotropy images which were then again cleaned from extracerebral tissue and CSF.

The main challenge facing voxel-based MTI analysis involves meeting the requirement for an optimal matching of the brains being compared. Therefore, a complex pre-processing procedure was employed as described before [38] consisting of the creation of a series of templates in order to derive the best possible parameter set for normalization. MTR maps were calculated according to the following formula: MTR = [(non-MT − MT)/non-MT] × 100. The pre-processing included the creation of a series of templates in order to derive the best possible parameter set for normalization. All PD-weighted scans were normalized to a scanner specific PD template, which was created from the studied sample population and subsequently smoothed with an 8-mm isotropic FWHM isotropic Gaussian kernel. The PD images in native space (non-normalized) were skull-stripped, segmented, multiplied with a binary mask and normalized to the previously skull-stripped PD-weighted template. This prevented any contribution of non-brain voxels, afforded optimal spatial normalization of the individual PD images and provided an optimized normalization parameter set for the PD images adapted to the investigated population sample. Because the segmentation of native images is performed on affine-normalized images, and because the probability maps used as Bayesian priors for segmentation are in stereotactic space, the optimized normalization parameter set was re-applied to the original PD-weighted images in native space. These optimally normalized PD images—now in stereotactic space—were again skull-stripped using the above described procedure. This resulted in optimally normalized PD images removed from extracerebral tissue and CSF. Finally, the optimized normalization parameter set was applied to the inherently co-registered MTR maps in native space and re-sliced with a final voxel size of 1 mm^3^. The normalized MTR images were then skull-stripped by applying the corresponding brain mask derived from the optimally normalized PD-weighted images. Analogous to the PD images, this resulted in optimally normalized MTR maps removed from non-brain structures. Images were smoothed to 8 mm using an FWHM Gaussian filter to improve signal-to-noise ratio.

#### Statistical analysis

Processed images of each tissue class were analyzed in the framework of the general linear model. This framework allows the testing, on a voxel-by-voxel basis, of the null hypothesis that the tissue volumes in the two populations (patients and controls) are the same. Group comparison of OCD patients and healthy controls was performed in SPM2 using ANCOVA. Only voxels exceeding an absolute threshold of 15 % were included in the analysis to minimize low signal-to-noise. Correlation with clinical parameters (YBOCS score) were done by using SPM multiple regression. Resulting statistical parametric maps of FA, ADC and MTR were derived at a significance level of *p* < 0.001, uncorrected with an extent threshold of 20 voxels. For regions where an effect was hypothesized, namely the fronto-striatal and limbic system (see introduction), a small volume correction (SVC) limited to the volume of that particular region was performed [39]. Here, we controlled for multiple comparisons by using the family wise error (FWE) method (*p* < 0.05). Significant voxel maxima were then converted from MNI space to Talairach space. Talairach coordinates were assigned to the anatomical structures by using Talairach Daemon [40, 41].

## Results

On visual inspection of the MR images no subject had focal atrophy of any brain region or movement artefacts which may have hindered alignment into standard space or segmentation into gray or white matter. Furthermore, there were no overt involuntary movements, which were monitored during scanning by one of the investigators (AG). Mean (+/− SD) intracranial volume did not significantly differ between patients with OCD and healthy controls.

### Group comparison

#### Diffusion tensor imaging

**Fig. 1:**
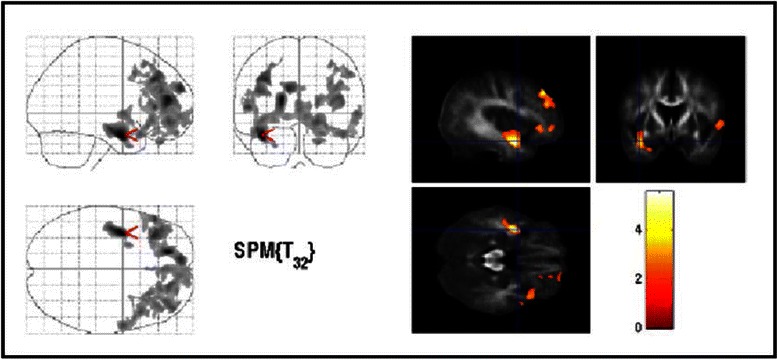
Areas of decreased regional fractional anisotropy in OCD patients compared with controls (thresholded at uncorrected *P* < 0.01, uncorrected, extend threshold 500 voxel). The three orthogonal planes on the left side represent a typical maximum intensity projection “glass brain”, and the set of images on the right side illustrates results superimposed on averaged and normalized FA images of the whole study population in selected planes. The images are shown in neurological convention. The colour bar represents the *t*-score. Cluster with local maxima were found near the area of the left ventral striatum. Moreover, we found reduced FA in the left medial frontal gyrus as well as in the OFC on both sides

Compared with healthy controls, patients with OCD showed reduced FA in the white matter of the left medial frontal gyrus as well as in the OFC on both sides (Fig. 1, Table 2). There were no significant increases of FA compared to healthy controls. In patients with OCD, we found increased ADC in the OFC on both sides and in the left middle temporal and parahippocampal regions (Table 2). No region demonstrated significant reduced ADC.

**Table 2:** MTR-, ADC- and FA-map differences in patients with OCD compared to healthy control subjects

Regions	Cluster size	*t*-value	MNI-space			*P*-value
			x	y	z	SVC
Decreased regional FA in OCD patients compared with controls						
Left sub-gyral WM	656	5.52	−39	−4	−19	0.001
Left medial frontal gyrus WM	472	4.95	−18	46	12	0.003
Left superior frontal gyrus WM	103	3.88	−11	58	22	0.035
Right inferior frontal gyrus WM	45	3.83	50	31	−9	0.038
Right superior temporal gyrus WM	75	3.77	60	−16	8	0.043
Right inferior frontal gyrus (BA 47) GM	74	3.97	56	22	−6	0.028
Right cuneus (BA 18) GM	88	4.10	13		10	0.021
Right postcentral gyrus (BA 3) GM	121	3.76	53	−17	39	0.044
Increased ADC-maps in patients with OCD compared with controls						
Left sub-gyral WM	166	4.02	−37	−1	−23	0.024
Left inferior frontal gyrus WM	99	4.00	−36	26	−5	0.025
Left middle frontal gyrus WM	77	4.36	−27	20	43	0.010
Left middle occipital gyrus WM	74	3.95	−46	−70	−4	0.026
Left parahippocampal gyrus WM	61	4.13	−29	−29	−18	0.019
Right superior frontal gyrus (BA 10) GM	931	5.08	21	66	11	0.002
Left middle temporal gyrus (BA 21) GM	70	4.02	−62	−2	−3	0.024
Left inferior frontal gyrus (BA 47) GM	33	3.74	−28	17	−13	0.043
Increased MTR maps in OCD patients compared with controls						
Left middle frontal gyrus	125	4.48	−39	24	43	0.008
Right inferior frontal gyrus (BA40)	58	4.46	51	25	5	0.008

Cluster size = number of voxels; x,y,z = Coordinates (in mm) of significant local maxima are given for information in MNI space (Montreal Neurological Institute, http://www.bic.mni.mcgill.ca). BA: Brodmann area. For regions where an effect was hypothesized a small volume correction (SVC) was performed. *P* values are given after family wise error (FWE) correction for the particular volume

### Magnetization transfer imaging

Compared to normal controls, patients with OCD displayed significant increases of MTR maps in the left middle frontal gyrus and GM of the right inferior frontal gyrus (Brodmann area (BA) 40). No significant decreases in MTR maps were found in OCD patients compared to healthy controls (Table 2).

### Correlations with Y-BOCS scores

#### Diffusion tensor imaging

**Fig. 2:**
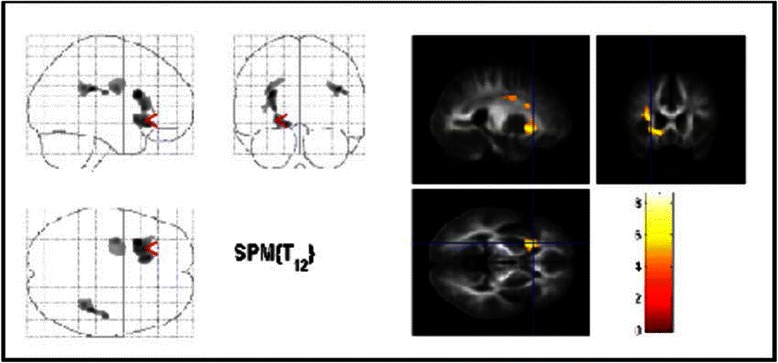
Areas with negative correlation between ADC-maps and Y-BOCS scores for compulsions in OCD patients. The same conventions apply as for Fig. 1. Significant voxels were found in the lentiforme nucleus, the striatum and the cingulate cortex on the left side (*p* < 0.001, uncorrected). Only cluster with more than 1400 voxels are shown for display purpose only

There were no significant correlations between ADC maps and Y-BOCS subscores for obsessions. We found significant negative correlations between the ADC maps and Y-BOCS subscores for compulsions in the left insula (BA13), the left nucleus lentiformis and striatum on the same side and WM of the cingulate cortex on the left side (Fig. 2, Table 3). The inverse correlation showed no significant results.

**Table 3:** Brain regions showing significant correlations with clinical Y-BOCS subscores for obsessions and compulsions

Region	Clusterlevel	*T*-value	MNI-space			*P*-value
			x	y	z	SVC
Negative correlation between ADC-maps and Y-BOCS scores for compulsions in OCD.						
Right sub-gyral	638	9.33	38	−1	−21	0.001
Left sub-gyral	4325	8.60	−30	16	18	0.001
Left anterior cingulate	547	7.41	−10	31	17	0.003
Left cingulate gyrus	257	6.20	−8	−36	31	0.010
Left lingual gyrus	347	6.00	−14	−68	−2	0.013
Left posterior cingulate	266	5.97	−3	−42	21	0.013
Left extra-nuclear	1637	5.96	−32	−6	25	0.013
Left lentiform nucleus	287	5.74	−11	−3	−2	0.017
Left insula (BA 13)	347	5.41	−31	−25	14	0.025
Positive correlation between MTI-maps and Y-BOCS scores for obsessions in OCD.						
Left inferior parietal lobule WM	138	7.48	−54	−42	44	0.005
Left middle temporal gyrus (BA 39) GM	1051	9.25	−49	−61	22	0.001
Left Cuneus (BA 18) GM	195	5.63	−2	−79	20	0.030
Left Cerebellum/Culmen	103	5.35	−4	−47	−9	0.039

Cluster size = number of voxels; x,y,z = Coordinates (in mm) of significant local maxima are given for information in MNI space (Montreal Neurological Institute, http://www.bic.mni.mcgill.ca). BA: Brodmann area. For regions where an effect was hypothesized a small volume correction (SVC) was performed. *P* values are given after family wise error (FWE) correction for the particular volume.

### Magnetization transfer imaging

**Fig. 3:**
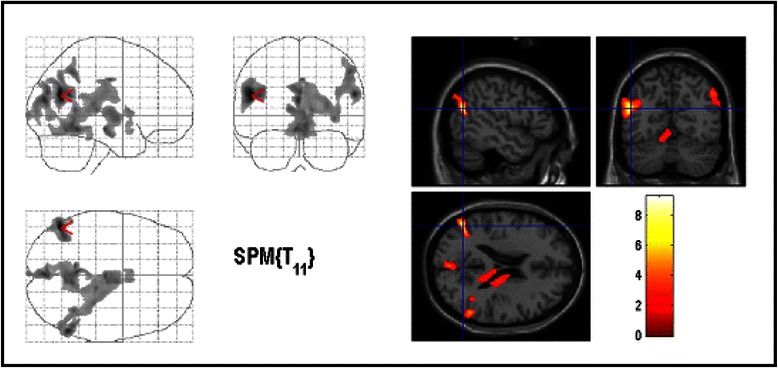
Areas with positive correlation between MTR-maps and Y-BOCS scores for obsessions. The significant results are superimposed on the average normalized MTR maps of the study population (thresholded at uncorrected *p* < 0.01 for display purpose only). The most significant voxels were found in the left parietal cortex (BA 39)

There were no significant correlations between MTR maps and Y-BOCS subscores for compulsions. Y-BOCS subscores for obsessions correlated positively with the MTR maps in the GM of the left parietal-temporal-occipital association cortex (BA 39), WM of the left inferior parietal lobule (Fig. 3, Table 3). The inverse correlation showed no significant results.

## Discussion

This is the first study using a multimodal approach with DTI and MTI in order to study structural brain changes in unmedicated, adult, male patients with OCD. Advanced imaging techniques may help to understand the complex relationship between biochemistry, structure and function in relation to each of the major symptom dimensions (obsessions and compulsions) of OCD. We therefore performed not only group comparisons between OCD-patients and healthy controls but additionally correlated MTR as well as ADC- and FA-maps with the Y-BOCS subscores for obsessions and compulsions to reassess the hypothesis that symptoms of OCD are mediated by partially distinct neural systems [6].

Three main findings emerged from this study. First, the group comparison between OCD patients and healthy controls demonstrated abnormalities of WM microstructure in multiple sites of the fronto-striatal system. Our patients showed decreased FA-maps in the OFC on both sides and increased diffusivity especially in both sides of the orbitofrontal parts of the brain (right superior frontal gyrus (BA 10)) and the left inferior frontal gyrus (BA 47) (Fig. 1). These structures have been established as “key brain regions” in the pathophysiology of OCD [3, 5, 27, 42, 43]. FA reflects directionality and coherence of water self-diffusion. Thus, tissues with highly regular fibers have high anisotropy, whereas those with less regular fibers, such as gray matter, have low anisotropy [19]. Therefore FA abnormalities in the aforementioned areas may reflect abnormalities in the myelin sheath and/or directional coherence of fiber tracts. Thus, our results support the prevailing hypotheses on the pathogenesis of OCD proposing that a defective [38], imbalanced [5], or hypertonic [44] striatum causes OCD symptoms by alterating the balance in cortico-striato-thalamo-cortical loops. It can be assumed that abnormalities in only one structure that belongs to this circuit can translate into changes in other parts of the loop or even in remote, but related brain regions [45].

The second main finding is a significantly negative correlation between the severity of compulsions (according to Y-BOCS scores) and ADC-maps in the left nucleus lentiformis and the striatum on the same side and WM of the left cingulate cortex. The ADC reflects the degree of apparent water diffusivity: tissues without obstacles such as cerebrospinal fluid have high water diffusivity, whereas those with obstacles such as WM have low diffusivity. ADC-maps, have recently been investigated in psychiatric disorders such as schizophrenia, mood disorders [46] and OCD [19]. Nakamae et al. [19] even suggested that in these conditions, ADC may be more sensitive to brain abnormalities than volume assessment. Therefore the ADC may be of particular value in the understanding of the pathology of OCD. Although the striatum is thought to be mainly involved in planning and modulation of movements [17], there is also substantial evidence for an involvement of the striatum in the generation of compulsions in OCD [3, 17, 47, 48]. Our finding of a negative correlation between Y-BOCS subscores for compulsions and the ADC-maps in the left ventral striatum therefore further corroborates this hypothesis. In line with our results, Fan et al. [17] showed that FA variables are positively correlated with the severity of compulsions in the left striatum. A further two MRI studies have reported a relationship between striatal volumes and the severity of OCD [3, 49]. To date, ADC, that is almost similar with mean diffusivity, has been investigated once in OCD [19]. However, in contrast to our results, Nakamae et al. [19] found no correlations between ADC and Y-BOCS scores. These inconsistent findings might be caused by different samples, small sample sizes and methodological differences. For example, Nakamae et al. [19] included only medicated patients, while we investigated unmedicated OCD patients. There are several ways to explain changes in ADC and there is also some uncertainty associated with such changes. One interpretation is that the ADC reflects the volume of the extracellular space or the degree of barriers to diffusion (tortuosity) for neurotransmitters due to perisynaptic glial processes and/or perineuronal membranes [50]. Therefore, ADC values could be a key parameter reflecting extrasynaptic neurotransmission.

In addition, we found a negative correlation between ADC maps and the severity of compulsions in the cingulate cortex. This negative correlation between ADC values and Y-BOCS compulsion scores seems to be contraintuitive at first sight as one would normally expect higher clinical scores to be related to a decrease in structural integrity. Nevertheless, one could assume that increased structure may favor a hypertonic state. Our results are in line with findings from structural and functional neuroimaging studies demonstrating abnormalities in the ACC [14, 45], but in contrast to other studies that failed to demonstrate changes in the FA in the ACC [22, 51, 52]. Although available data are contradictory regarding the role of the cingulate cortex in the pathogenesis of OCD, dysfunctional networks have been suggested to be involved in the pathogenesis of OCD including not only the OFC and the motor cortex, but also the ACC and the rostral cingulate motor area [7]. Furthermore, the anterior and the posterior cingulate cortices are major components of the cortico-striato-thalamo-cortical circuit [53]. Finally, the cingulum bundle, a major association fiber tract that interconnects limbic structures, has been shown to be involved in OCD pathology [51, 52].

The third striking finding of this study is a significantly positive correlation between the severity of obsessions and the MTR in the left parietal cortex (BA 39). To our best knowledge, this is the first study using MTI in patients with OCD. We decided to use MTI, since this method is said to be more sensitive to subtle structural brain changes than conventional volumetric imaging [30]. MTI is a nuclear magnetic resonance technique relying on the transfer of energy between highly bound protons within structures as myelin and the very mobile protons of free water. On brain, the major macromolecules in the bound proton pool are cell membrane proteins and phospholipids in gray and white matter and myelin in white matter. Bound protons which are undetected by conventional MRI because of their very short relaxation times, can be preferentially saturated using an off-resonance radio frequency pulse. This leads to a reduction in signal intensity, which is dependent on macromolecular density. These changes can be quantified by the MTR. MTR correlates with in vivo measurements of N-acetyl-aspartate, a marker of neuronal integrity [54], and MTR reductions correlate with myelin and axonal loss in the white matter in post mortem tissue, and in vivo in a range of neurological diseases [55]. In the present study, however, patients showed an increase in MTR in several regions with could be due to the development of myelinisation. In addition to fronto-striatal and limbic regions, increasing attention has been paid to an involvement of the parietal cortex in the pathophysiology of OCD [2, 12, 14, 20, 43]. Accordingly, structural [14, 20, 51, 56], resting state [57] and activation [58] neuroimaging studies found abnormalities in this brain region. It is also intriguing that studies of WM abnormalities in OCD have reported abnormalities in bilateral supramarginal gyri [20] and parietal white matter [56]. Since the parietal cortex is thought to be involved in attention, visuospatial processes [59], various executive functions [60], and working memory [61], parietal dysfunction may also contribute to cognitive impairment in OCD [2]. Furthermore, the parietal lobe has been specifically implicated in planning and response inhibition, functions that have also been reported to be impaired in OCD [62]. According to Valente et al. [14], parietal lobe dysfunction may interact with fronto-subcortical circuitry through the direct anatomical connections between associative parietal areas and some of the key regions implicated in OCD, including the lateral orbitofrontal cortex, the striatum and the mediodorsal thalamic nucleus.

The following limitations of our study have to be addressed. Firstly, the voxel-by-voxel analysis used was originally intended for the use in large samples and requires smoothing of the images with loss of resolution for small structures. Furthermore, the large number of comparisons required corrections for multiple comparisons. This might explain why we did not find significant MTR differences between OCD patients and healthy controls on a whole brain analysis. Moreover the small size of our group makes the results vulnerable to type I or type II errors, although in recent DTI and MTI studies a similar number of patients was suitable to detect regional differences compared to normal controls [19, 22]. The locations of the MTI and DTI abnormalities overlapped in some regions but were not identical. This apparent inconsistency could be due to the use of a volume of interest that - while increasing the power of the analysis by reducing the number of comparisons - may exclude potentially abnormal areas.

## Conclusions

This is the first study using MTI to investigate structural brain abnormalities in OCD patients. In addition we analyzed not only group comparisons between OCD patients and healthy controls but also obsession and compulsion subscores (Y-BOCS) separately using both DTI and MTI in a carefully selected group of adult, unmedicated, male OCD patients without comorbidities and age- and sex-matched normal controls. In our study, we show a positive correlation between MTR and Y-BOCS obsession scores with an increased integrity of tissue structure in the left parietal cortex, including myelination and axonal density reflected by the MTR which was used for the first time in our study. Y-BOCS subscores for compulsions correlated negatively with ADC maps in the left striatum, the left nucleus lentiformis and the left cingulate cortex. Thus, our results support the hypothesis that OCD is characterized by widespread cerebral changes which underlines the heterogeneity of this disorder.
